# Fair Data, Bayesian Statistics and Human Cohort Studies: Current Trends in Metabolomic Research

**DOI:** 10.3390/metabo14110576

**Published:** 2024-10-25

**Authors:** Oliver Fiehn

**Affiliations:** West Coast Metabolomics Center, University of California, 451 Health Sciences Drive, Davis, CA 95616, USA; ofiehn@ucdavis.edu

This Special Issue was published to celebrate 10+ years of research and services at the UC Davis West Coast Metabolomics Center (WCMC). The WCMC was founded based on long-standing metabolic research in Davis, first by Professor Bruce Hammock on oxylipins and later joined by Professor Oliver Fiehn (untargeted metabolomics), adjunct Professor John Newman (lipid mediators) and Professor Ameer Taha (lipids in brain development). Together, they formed the research core of the NIH-funded regional center in metabolomics (2012–2023), contributed to its continuing monthly seminar series and assisted in our professional training courses in metabolomics (1-week, 2-week and ten 4-h courses per year). Importantly, validated analytical and cheminformatics methods from these research laboratories were then incorporated into the sizable WCMC service core that operates solely on fee-for-service projects. Using 16 mass spectrometers, more than 300 service projects have been completed each year, accounting for more than 30,000 samples. These service projects continue to fund the backbone of the WCMC, its suite of cheminformatics tools including MassBank of North America, BinBase and the Chemical Translation Service and ancillary software that we developed with visiting scientists, specifically Professor Hiroshi Tsugawa (Japan, on MS-DIAL and MS-FINDER). Hence, while NIH funding for the center has concluded as planned for the 10-year funding period in NIH Common Funds projects, the WCMC services, training and outreach activities continue. Research has progressed on dedicated projects in each of the four research laboratories. Here, we present improvements in metabolomics methods but also typical applications in both WCMC research laboratories and in biological projects that received WCMC service data.

Five method contributions cover the different aspects in the MS-based metabolomics workflow. Contribution 01, by Bremer and Fiehn (2023), provided a novel and critical aspect of FAIR data re-use in metabolomics databases: How can we capture the biological relevance of metabolomics projects? The authors presented a different approach to the conundrum of classic Omics databases that usually include raw and result data but present limited biological metadata. For the public, this lack of biological metadata renders metabolomics results difficult to interpret for a single study or even to compare results between studies. More importantly, while typical biological studies use one matrix (such as plasma or liver) and one species (such as mouse), the extent to which the samples reflect the impact of interventions (such as drugs) and time points is lost. This information must be given on the level of samples and not merely described in an overall study abstract. The authors’ tool, SMetaS, is the first instance of such a sample-based metadata standardizer. In Contribution 02, Cajka et al. (2023) explored the impact of the quality of solvents in LC-MS based data acquisitions. For lipidomics, sources of isopropanol were found to include a surprisingly high amount of contamination that led to ion suppression and large differences in the abundance of background ions. They identified the use of plasticizers (dioctyl phthalate), antioxidants (Irganox 1076), polymer additives (Irgafos 168) and other chemicals in supposedly pure isopropanol solvents. The amount of these contaminants was irrespective of the price of the LC solvents, with the most expensive solvent labeled as ‘unacceptable’ by the authors. Contribution 03, by Wang et al. (2023), focused on another important issue in metabolomics, compound annotation. While compound annotations have largely improved in accurate mass LC-MS/MS over the past 10 years, de novo identifications in GC-MS have remained rare. Here, the problem is that the GC-MS usually performs hard electron ionization at -70 eV, which yields highly reproducible mass spectra but little or no abundance of the intact molecule itself. Hence, unknown compounds in the GC-MS are difficult to identify. Here, the authors showcase the use of softer chemical ionization with methane as the reagent gas and subsequent software that automatically assigns the molecular ion from a series of reactant molecule adducts. In Contribution 04, a massive compendium is presented by Zhang et al. (2023a) tackling multiple approaches to data normalization in GC-MS based metabolomics. They first compared the different sialylation derivatization reagents against chloroformate with respect to the metabolite coverage, precision and accuracy. For plasma metabolomics, the precision and accuracy improved when internal standards were used for specific amino acids; however, surprisingly, using the sum abundance of all the internal standards did not yield much better precision on the relative standard deviation of all the identified metabolites than normalizing to the classic sum parameters (without internal standards). This observation led the authors to assume that GC-MS, in comparison to LC-MS, may be subject to additional stochastic factors that decrease the quantitative precision. They presented a new algorithm that addresses such stochastic factors, the denoising autoencoder. The authors showed that with this data normalization, very large human cohort studies with more than 4000 samples yielded a remarkably low median precision of <20% RSD in independent quality controls, better than six other types of data normalization methods. Contribution 05 concluded the methods section of this Special Issue of the UC Davis West Coast Metabolomics Center. Here, Brydges et al. (2023) showcased that Bayesian statistics readily overcomes the problems of classic (‘frequentist’) statistical approaches that plague metabolomic studies. In frequentist statistics, all studies are regarded as independent from each other. In Bayesian statistics, each published study informs expectations for subsequent projects, continuously learning and narrowing down the most likely factors that underlie a specific disease. Such statistical approaches are utilized in many areas of daily life, from weather forecasts to economics, but they are much rarer in biology and medicine. The authors show that Bayesian statistics leads to a better interpretation of the biological meaning and impact of metabolomic data, because these data do not need to be confined to multiple testing corrections. Instead, Bayesian results display the likelihood of one hypothesis (e.g., the dysregulation of a metabolite in a case/control study) against the null hypothesis (no metabolic difference). By combining data from three studies in chronic fatigue, the authors pinpoint damage in peroxisome function as a likely contributor to the disease.

The Special Issue then continues by highlighting metabolic differences in seven different biological and biomedical projects. Contribution 06, by Cumeras et al. (2023), highlighted the vast differences in stool metabolites between people who eat meat (omnivores) and those who only eat plant-based foods (vegans). This material was established by the National Institute of Standards and Technology (NIST), an authoritative agency in the USA. With this collection average that originated from a wide range of individuals, NIST intends to establish ‘standard fecal matter’ that can be distributed to all laboratories as reference material, as a yardstick to compare results from future studies. In a workshop, the NIST had previously asked test laboratories, including the WCMC, to list the top-20 metabolic differences between vegan and omnivore stool and between freeze-dried and aqueous reference fecal matter. Cumeras reports that many hundreds of metabolites were either absent or present in the vegan versus omnivore comparison, pointing to the huge differences dietary habits have on both microbiome and metabolome compositions in the large intestine. Contribution 07 by Zhang et al. (2023b) investigated links between genetics and metabolomics in a different way. The Knockout Mouse Project (KOMP) was initiated in 2006 to develop knockout mutations for every protein-coding gene in the mouse genome, linking the function of mouse genes to a large array of phenotyping assays in conjunction with the International Mouse Phenotyping Consortium (IMPC). While 108 different phenotypes were recorded for more than 7000 mouse gene knockouts, the impacts of such knockouts on the metabolome were unknown. Aligned with a proteomics study, Zhang et al. found that all 30 tested gene knockouts, including 10 enzyme knockouts, showed profound differences in mouse plasma metabolomes. Interestingly, most genes also showed large differences between female and male mice, for example, for Sra1 that regulates the signaling pathways involved in breast cancer. Similar observations were found in peroxisomal Phyh (Refsum disease), Npc2 (Niemann–Pick disease), Mfap4 (chronic obstructive pulmonary disease) and others. Mouse gene alterations were also the focus in Contribution 08 by Wen et al. (2023). Wen studied the impact of the Slc19a3 intestinal thiamine transporter in knockouts and in human SLC19A3 transgenic mice. By using different thiamine-supplemented diets, Wen et al. found reduced levels of nucleic acid and amino acid derivatives in the mouse brain when thiamine transport was impaired. This disease focus of metabolomics studies in neurological conditions was followed up in Contribution 09, by WCMC guest scientist and Fulbright scholar Dahabiyeh, (Dahabiyeh et al. 2023). Dahabiyeh showed robust plasma metabolic signatures when studying a human cohort of subjects diagnosed with Parkinson’s disease (PD), which currently has an unclear etiology, a lack of definite diagnostic tests and no effective treatments. In comparison to age-matched healthy controls, PD patients showed highly significant differences in 169 plasma lipids. A panel of ten significantly altered circulating lipids (mostly sphingo- and phospholipids, including lipids with odd-chain fatty acyls) resulted in a strong receiver operating characteristic curve with an AUC = 0.974 that could lead to a diagnostic test in the future, once validated in subsequent studies. In a second human cohort study, Contribution 10, by Jess et al. (2023), reported on recurrent *C. difficile* infection in a pediatric population. The authors found a significant increase in short chain fatty acids after fecal microbiota transplantation compared to prior intervention in the same subjects. The increase in acetate, propionate and isovalerate levels lasted for at least 12 months after the microbiota intervention and effectively counteracted the decreased levels of these microbial metabolites that are usually associated with *C. difficile* infections. In Contribution 11, de Oliveira et al. (2023) showcased the use of metabolomic services rendered by the WCMC. The authors tested metabolomic changes in the vaginal discharge of dairy cows as biomarkers to predict metritis development, an inflammation of the uterus with a striking incidence of >20% in cows. Specific metabolites were discovered to be associated with cows cured using ceftiofur treatment. These metabolites may lead to biomarker tests to predict the onset, progression and amelioration of dairy cow metritis.

These contributions showcase the large improvements that MS-based metabolomics has achieved over the past 10 years but also challenges that lie ahead.

1. No contribution stated that metabolomics is an emerging field. Metabolomics has matured and is now routinely applied in various disciplines from population health to cell biology.
2. A sign of maturity is how intricate new developments have become. Former studies focused on data processing and enumerating all detected signals [1], often without significant efforts in compound identifications. Today, it is unthinkable for metabolomics studies not to disclose the number of annotated compounds in supplements or database uploads [2,3].
3. Publication of data from corporate metabolomic service providers remains problematic, as long as the raw data are not publicly available to scrutinize the compound annotations [4].
4. Tools in compound annotation have become more sophisticated [5]. Initially, database propagations were deemed sufficient [6]. Today, critical voices raise the issue of confidence in metabolome annotations, specifically if they rely only on mass spectra and do not consider additional lines of evidence such as retention times and the complexities of in-source fragments and adducts [7,8].
5. Often, unidentified signals are not even reported anymore, as it is difficult to track such signals between assays and laboratories [9,10]. Many studies, including some of the contributions published in this Special Issue, focus solely on the biological implications and conclusions.
6. This progress means that future developments must focus on three aspects:
  (a) Make metabolomics more quantitative, to enable comparisons across studies.
  (b) Make metabolomics more publicly accessible, to track unidentified metabolites across studies and gain trust in compound annotations.
  (c) Make metabolomics more interpretable, to find novel biological functions for metabolites.

In combination, these tasks present opportunities rather than insurmountable challenges. Large databases such as MASST can introduce new avenues for the large-scale comparisons of data across studies [11]. Consortia and international collaborations provide chances for data exchange, for example, in mass spectral libraries. In addition, the improved description of biological context and study metadata will lead to better interpretation of metabolic alterations. Importantly, better tools and databases will improve the specificity of biological conclusions. Generic statements on the supposed dysregulation of aminoacyl-tRNA pathways [12,13] that authors have used straight from MetaboAnalyst assignments will then be a matter of the past.

## Data Availability

Data are contained within the article.
